# Optical Redox Imaging Differentiates Triple-Negative Breast Cancer Subtypes

**DOI:** 10.1007/978-3-030-48238-1_40

**Published:** 2021

**Authors:** Jinxia Jiang, Min Feng, Annemarie Jacob, Lin Z. Li, He N. Xu

**Affiliations:** Britton Chance Laboratory of Redox Imaging, Department of Radiology and Johnson Research Foundation, Department of Biochemistry and Biophysics, Perelman School of Medicine, University of Pennsylvania, Philadelphia, PA, USA

**Keywords:** NADH and FAD, Intrinsic fluorescence, Redox plasticity, Triple-negative breast cancer, Metabolic characterization

## Abstract

Triple-negative breast cancer (TNBC) is a highly diverse group of cancers with limited treatment options, responsible for about 15% of all breast cancers. TNBC cells differ from each other in many ways such as gene expression, metabolic activity, tumorigenicity, and invasiveness. Recently, many research and clinical efforts have focused on metabolically targeted therapy for TNBC. Metabolic characterization of TNBC cell lines can facilitate the assessment of therapeutic effects and assist in metabolic drug development. Herein, we used optical redox imaging (ORI) techniques to characterize TNBC subtypes metabolically. We found that various TNBC cell lines had differing redox statuses (levels of reduced nicotinamide adenine dinucleotide (NADH), oxidized flavin adenine dinucleotide (FAD), and the redox ratio (FAD/(NADH+FAD)). We then metabolically perturbed the cells with mitochondrial inhibitors and an uncoupler and performed ORI accordingly. As expected, we observed that these TNBC cell lines had similar response patterns to the metabolic perturbations. However, they exhibited differing redox plasticity. These results suggest that subtypes of TNBC cells are different metabolically and that ORI can serve as a sensitive technique for the metabolic profiling of TNBC cells.

## 40.1 Introduction

Triple-negative breast cancer (TNBC) is characterized by the lack of expression of estrogen receptor (ER), progesterone receptor (PR), and human epidermal growth factor receptor 2 (HER2) [1]. This heterogeneous group of tumors constitutes about 15% of all breast cancers and is generally more aggressive, recurrent, and metastatic than other breast cancers [2]. Various studies have investigated the underlying genomic differences in TNBC, and results suggest further characterization by subtyping [3, 4]. One established method of characterization is by analyzing gene expression profiles [4]. This method categorizes TNBC cell lines into the following subtypes: basal-like, immunomodulatory, mesenchymal, mesenchymal stemlike, and luminal androgen receptor. However, altered metabolism is also a hallmark of cancer and can potentially be used in the characterization of TNBC subtypes.

The mitochondrial redox status provides critical information about the energy-linked biological processes in cells and tissues for various types of cancer, including TNBC [5]. Nicotinamide adenine dinucleotide (NADH) and oxidized flavin adenine dinucleotide (FAD) are two coenzymes that play important roles in the mitochondrial electron transport chain (ETC). Pioneered by Chance et al. [6, 7], the redox ratio, calculated by taking the ratio of measured endogenous fluorescence of NADH and FAD, can be used as an indicator of the mitochondrial redox state. Since then, optical redox imaging (ORI) techniques have demonstrated increasing applications in cancer research, such as differentiation among cancer aggressiveness [8–10] and monitoring therapeutic effects [11, 12]. In the present study, we report the use of ORI as a tool in differentiating the mitochondrial redox status among four TNBC cell lines (MDA-MB-231 and MDA-MB-436 mesenchymal-like, MDA-MB468 basal-like 1, HCC1806 basal-like 2) and characterizing their redox plasticity under metabolic modulations. Such characterizations could provide useful information for the metabolic stratification of TNBC cell lines.

## 40.2 Methods

### 40.2.1 Cell Culture and Perturbation Drugs

TNBC cell lines MDA-MB-231, MDA-MB-436, MDA-MB-468, and HCC1806 were cultured in RPMI 1640 (Gibco^®^, Cat No. 11875085) supplemented with 10% fetal bovine serum (Corning^®^). Cells were maintained at 37 °C and 5% CO_2_ and passaged at 80% confluency using 0.25% trypsin-EDTA. Oligomycin (oligo), trifluoromethoxy carbonylcyanide phenylhydrazone (FCCP), rotenone (rot), and antimycin A (aa) (Sigma^®^) were reconstituted in DMSO (Sigma^®^) and used at the following final concentrations: oligo (2 μg/mL), FCCP (0.5 μM), rot (1 μM), and aa (1.25 μg/mL).

### 40.2.2 Live Cell Optical Redox Imaging and Analysis

As detailed previously [11], 80,000 cells were seeded on 35 mm glass bottom dishes (MatTek^®^) and incubated at 37 °C and 5% CO_2_ overnight. Approximately 1 hour before imaging, the medium was replaced with 1 mL live cell imaging solution (Life Technology^®^) supplemented with 11 mM glucose and 2 mM glutamine (Sigma^®^), the same as in the RPMI 1640 medium. A DeltaVision widefield microscope was used to collect NADH and FAD fluorescence signals. The excitation band-pass filters for NADH and FAD channels were 360/40 nm and 470/40 nm, respectively; the emission filters of NADH and FAD channels were 455/50 nm and 520/40 nm, respectively. Nine to fifteen dishes were imaged on different days for each cell line with three fields of view (FOV) for each dish with an exposure time of 3 seconds for each channel. For mitochondrial redox plasticity experiments, perturbation drugs were added sequentially in the order of oligo, FCCP, and a mixture of rot and aa (rot/aa), allowing the cells to be pushed to the two redox extremes and the consequent calculation of the cell lines’ redox plasticity. The dishes were imaged 3–5 minutes after each drug administration. A customized MATLAB^®^ program was used to extract NADH and FAD signals and the pixel-to-pixel based FAD/(NADH+FAD) ratio, which were first averaged across FOVs and then across individual dishes to obtain the group mean and standard error of the mean (SEM). Heteroscedastic two-tailed t-tests were used for statistical analyses of redox differences among cell lines and the differences before and after a specific treatment. *P* < 0.05 was considered statistically significant.

## 40.3 Results and Discussion

### 40.3.1 ORI Differentiates TNBC Cell Lines

**Fig. 40.1:**
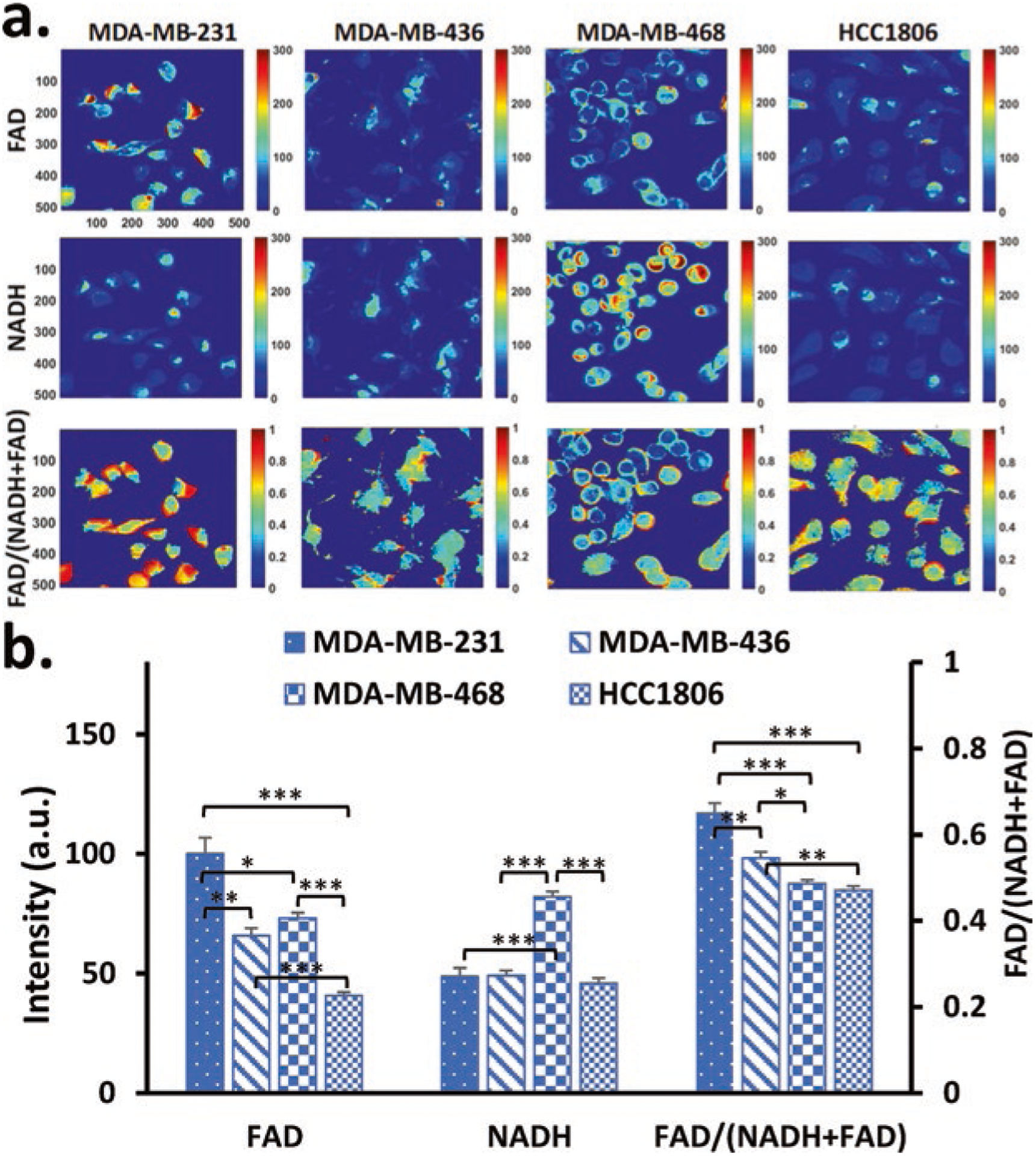
ORI differentiates TNBC cell lines. (**a**) Typical redox images of MDA-MB-231, MDA-MB-436, MDA-MB-468, and HCC1806. FAD and NADH color bar indicates signal intensity in arbitrary unit. FAD/(NADH+FAD) color bar indicates ratio range from 0 to 1. (**b**) Quantification of FAD, NADH fluorescence intensities, and FAD/(NADH+FAD) ratio. Primary y-axis quantifies FAD and NADH signal intensity, and secondary y-axis quantifies the FAD/(NADH+FAD) ratio. Results are reported as mean + SEM. *p < 0.05, **p < 0.001, ***p < 0.0001

We first imaged MDA-MB-231, MDA-MB-436, MDA-MB-468, and HCC1806 cells under normal conditions (Fig. 40.1a). Our quantitative analysis shows MDA-MB-231 exhibiting the highest FAD intensity and MDA-MB-468 exhibiting the highest NADH intensity (Fig. 40.1b). FAD intensity was significantly different (*p* < 0.05) between all cell lines except between MDA-MB-436 and MDA-MB-468. NADH intensity was similar in MDA-MB-231, MDA-MB-436, and HCC1806. Furthermore, we found that MDA-MB-231 exhibits the highest redox ratio of 0.65 ± 0.02, followed by MDA-MB-436, MDA-MB-468, and HCC1806, with the redox ratios of 0.55 ± 0.01, 0.49 ± 0.01, and 0.47 ± 0.01, respectively. The TNBC cell lines significantly differ in their redox ratios, with an exception between MDA-MB-468 and HCC1806, both of which are classified as the basal-like subtype (*p* > 0.05).

### 40.3.2 ORI Detects Metabolic Changes in TNBC Cell Lines

**Fig. 40.2:**
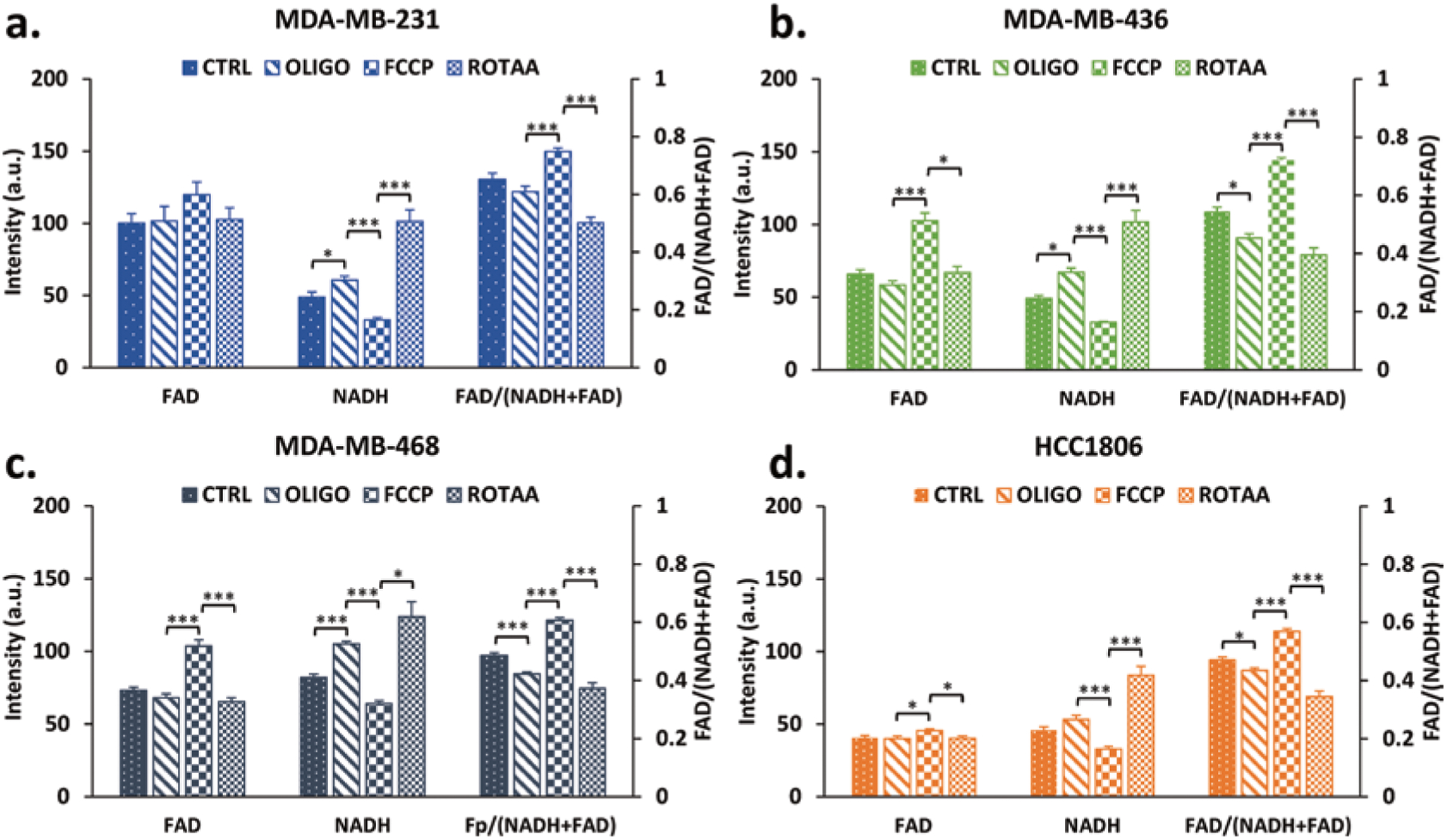
ORI detects metabolic changes in TNBC cell lines. Quantification of FAD, NADH fluorescence intensities, and FAD/(NADH+FAD) ratio for (**a**) MDA-MB-231, (**b**) MDA-MB-436, (**c**) MDA-MB-468, and (**d**) HCC1806 under control conditions, after sequential additions of oligo, FCCP, and rot/aa. Results are reported as mean + SEM. *p < 0.05, ***p < 0.0001

To show that ORI can reflect the metabolic phenotypes of TNBC cell lines, we sequentially administered oligo, FCCP, and rot/aa and obtained the NADH and FAD fluorescence signals accordingly. Oligo is an inhibitor of the ATP synthase in the ETC, causing a buildup of NADH. FCCP is an uncoupling agent that collapses the proton gradient and disrupts the mitochondrial membrane potential, causing unregulated oxidation of NADH and FADH_2_ to NAD^+^ and FAD, respectively, resulting in decreased NADH and increased FAD. Conversely, rot and aa are inhibitors of complexes I and III, respectively, fully inhibiting respiration, resulting in a maximal NADH signal and decreased FAD. Our results show that FCCP and rot/aa treatments effectively caused a decrease and increase in NADH intensity, respectively, in all four cell lines and an increase and decrease of FAD in three lines except MDA-MB-231 (Fig. 40.2). Furthermore, FCCP and rot/aa also resulted in a significant change in the redox ratios of all four cell lines and induced a more oxidized and a more reduced redox state, respectively. Although oligo administration did induce some significant changes at least in one of the redox indices (FAD, NADH, or the redox ratio) in each cell line, it was not as consistent as those observed from FCCP and rot/aa administration.

### 40.3.3 TNBC Cell Lines Have Differential FAD and Redox Ratio Plasticity

**Fig. 40.3:**
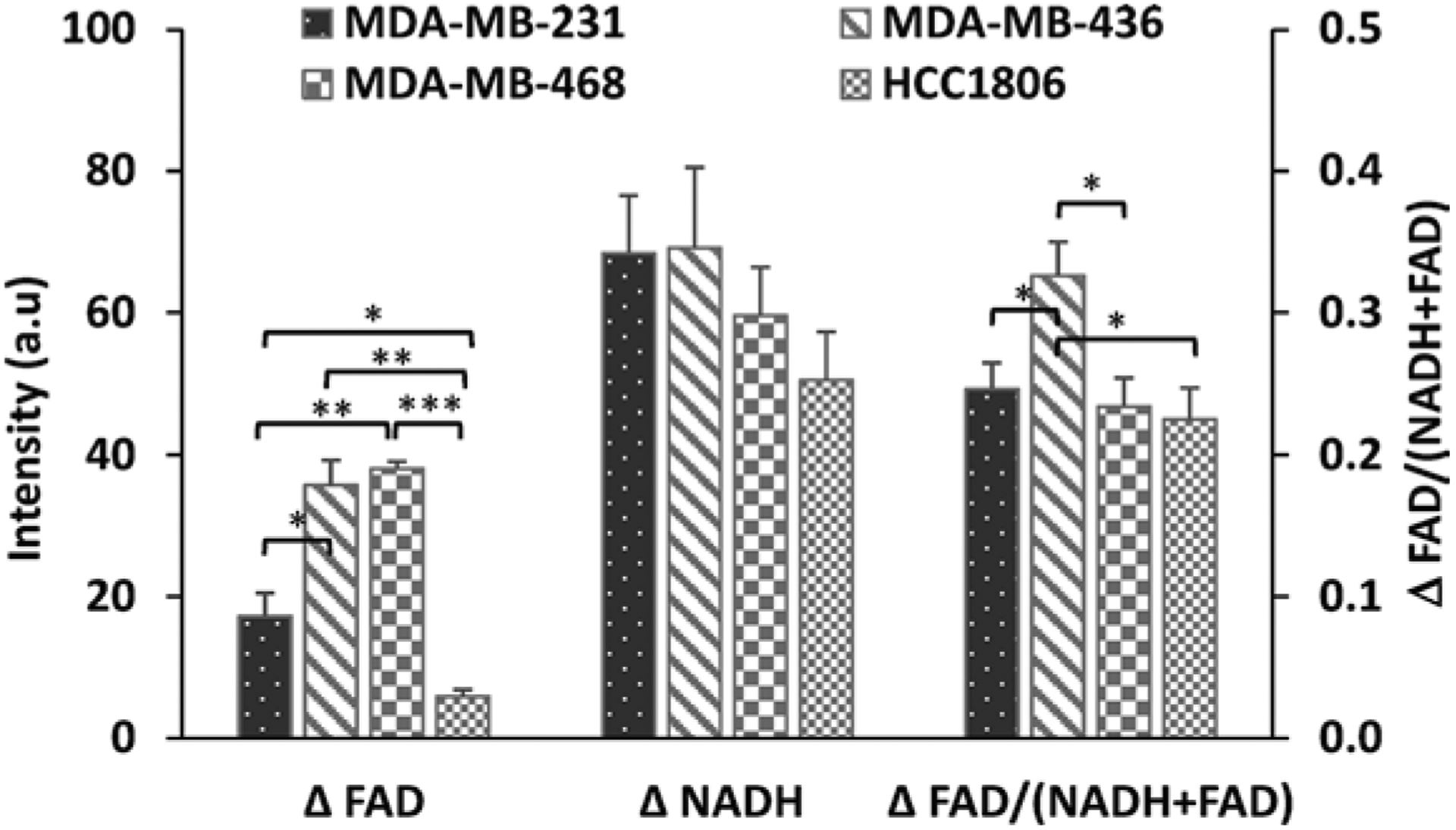
TNBC cell lines have differential redox plasticity. Quantification of FAD, NADH, and redox ratio plasticity. Results are reported as mean + SEM. *p < 0.0 5; **p < 0.001, ***p < 0.0001

By taking the difference between the average fluorescence values after FCCP and rot/aa application, we quantified the changes, i.e., the plasticity in NADH, FAD, and the redox ratio, in the TNBC cell lines [11]. Our results showed significant variations in FAD plasticity between the cell lines (Fig. 40.3). MDA-MB-436 and MDA-MB-468 had similar FAD plasticity values of 36 and 38 a.u., respectively, followed by MDA-MB-231 at 17 a.u. and HCC1806 at 6 a.u. No significant differences were found for NADH plasticity across cell lines. Our results also showed that MDA-MB-436 had the largest change in redox ratio, valued at 0.33 a.u., and MDA-MB-231, MDA-MB-468, and HCC1806 had similar changes in redox ratio (0.25, 0.23, and 0.23, respectively).

To our best knowledge, this is the first systematic investigation that reports how ORI differentiates the redox plasticity of TNBC cells. Changes in the redox indices caused by therapeutic interventions could reflect compromised mitochondrial redox plasticity. This information would be helpful in assessing drug effects in vitro, such as screening drugs for developing therapeutic interventions that target the metabolic activities in TNBC cells. Our previous studies have linked the redox ratio of the more oxidized tumor subpopulations to breast tumor and melanoma metastatic risk [9, 10]. Therefore, redox plasticity can also be further investigated to assist in the prediction of different metastatic potential of the TNBC tumors.

In short, the four TNBC lines can be clearly differentiated by ORI, and the underlying mechanisms remain to be elucidated.
